# Single-cell and spatial transcriptomics reveals the key role of *MCAM*^+^ tip-like endothelial cells in osteosarcoma metastasis

**DOI:** 10.1038/s41698-025-00896-8

**Published:** 2025-04-13

**Authors:** Haijun Tang, Yangjie Cai, Mingxiu Yang, Shengping Tang, Qian Huang, Hening Li, Shangyu Liu, Hongcai Teng, Tianyu Xie, Maolin He, Yun Liu, Shijie Liao

**Affiliations:** 1 Department of Spine and Osteopathic Surgery, The First Affiliated Hospital of Guangxi Medical University, Nanning, Guangxi China; 2 Department of Traumatic Orthopedic and Hand Surgery, The First Affiliated Hospital of Guangxi Medical University, Nanning, Guangxi China; 3 Department of Oncology, The First Affiliated Hospital of Guangxi Medical University, Nanning, Guangxi China

**Keywords:** Bone cancer, Oncogenes

## Abstract

Osteosarcoma, the most common primary malignant bone tumor in children and adolescents, is highly aggressive and prone to metastasis. Endothelial cells (ECs) are involved in angiogenesis and play a key role in promoting the metastasis of tumor. However, research on tip-like ECs within osteosarcoma was extremely rare. In this study, a single-cell atlas of ECs was constructed using single-cell transcriptomic data. It was found that tip-like ECs were abundant in the primary tumors and metastatic foci. Gene sets score analysis indicated their enrichment in pathways associated with angiogenesis and metastasis. What’s more, *MCAM* was highly expressed in tip-like ECs and was likely to promote the metastasis of osteosarcoma. *MCAM* was also found to be highly expressed in the ECs of metastatic lymph nodes when compared to normal lymph node samples. Meanwhile, spatial transcriptomics data confirmed the presence of *MCAM-*positive ECs in metastatic lymph node, closely localized to osteoblasts. In vitro assays, including qRT-PCR, tube formation, and immunofluorescence, validated the role of the *MCAM* gene in promoting angiogenesis. In conclusion, tip-like ECs may promote tumor metastasis by enhancing angiogenesis. *MCAM* was a functional gene for tip-like ECs and could serve as a target for the treatment of osteosarcoma.

## Introduction

Osteosarcoma (OS), the most common primary bone tumor in children and adolescents^1,2^, is highly invasive^3^. The five-year survival rate for patients with localized disease is approximately 60%, but drops to 20% in cases of metastasis^1^. The mechanisms underlying osteosarcoma metastasis are complex and regulated by multiple factors. Tumor metastasis is a dynamic process in which tumor cells initially detach from the primary site, invade surrounding blood and lymphatic vessels, and subsequently entering the circulation to form circulating tumor cells (CTCs)^4^. CTCs may eventually colonize tissue or organs, such as the lymph nodes and lungs. Therefore, gaining insight into the metastatic mechanisms is crucial for improving the prognosis of osteosarcoma patients.

Endothelial cells (ECs), a significant component of the tumor microenvironment, are pivotal in tumor growth and metastasis^5^. ECs contribute to angiogenesis, facilitating the formation of blood vessels that supply nutrients essential for tumor expansion and metastasis^6^. Additionally, ECs directly regulate tumor growth by secreting angiocrine factors such as growth factors, adhesion molecules, and chemokines^7^. Research has also shown that the ECs play a crucial role in osteosarcoma metastasis^8,9^. For example, Ling et al.^10^ demonstrated that ECs could secrete Von Willebrand factor (*VWF*), which promoted epithelial-mesenchymal transition (EMT) and metastasis of osteosarcoma. However, the heterogeneity of ECs within tumors is evident, with different EC subtypes potentially exerting distinct roles^11^. Therefore, dissecting the heterogeneity of EC is crucial for elucidating the metastatic mechanism of tumor.

Single-cell RNA sequencing (scRNA-seq) allows the detection of gene expression profiles at the single-cell level, making it a powerful tool for dissecting EC heterogeneity^12,13^. For instance, He et al.^14^ used scRNA-seq to dissect the functional heterogeneity of EC in lung metastatic lesions of osteosarcoma. In another study, Wei et al.^9^ revealed that TYROBP-positive ECs may promote tumor progression by interacting with malignant cells and modulating immune cell infiltration. However, comprehensive exploration of EC heterogeneity in osteosarcoma remains limited.

In the present study, scRNA-seq and spatial transcriptomics were integrated to explore the heterogeneity landscape of ECs in osteosarcoma. A novel subgroup of EC (*MCAM*^**+**^ tip-like ECs) was identified, and the crucial role of *MCAM* in ECs was validated through qRT-PCR, tubule formation assays and immunofluorescence. These findings provided a rich and innovative preliminary foundation for further functional studies on ECs in osteosarcoma.

## Results

### Construction of osteosarcoma cell atlas

**Fig. 1:**
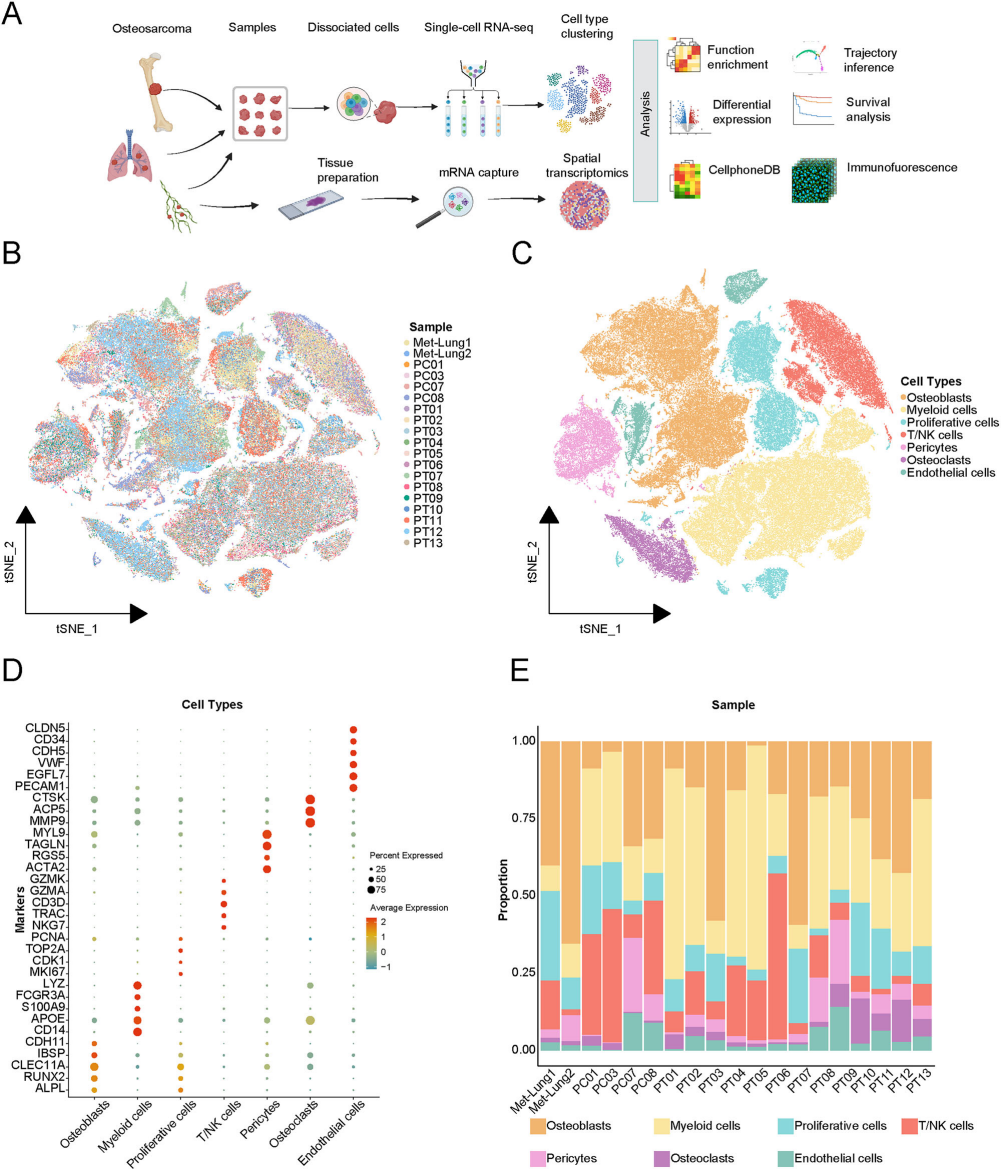
Single-cell atlas of osteosarcoma. **A** The overall flowchart of the research (Created in BioRender.com). **B** The tSNE plot of the multicellular ecosystem of 19 tissue samples. **C** tSNE plot of 7 cell clusters in the multicellular ecosystem. **D** The marker genes of each cell cluster. **E** Composition of cell clusters in different samples.

Sample information and corresponding groupings were as follows: 13 osteosarcoma primary tumor (PT) samples (including 6 patients without chemotherapy and 7 patients who underwent chemotherapy), 2 lung metastasis samples (Met-Lung), and 4 paracancerous (PC) samples (Fig. 1A, B). A single-cell atlas of osteosarcoma was constructed based on the above data, incorporating a total of 129,315 high-quality cells. Cells were categorized into 7 distinct clusters based on representative classification markers: osteoblasts (*CDH11*, *IBSP*, *CLEC11A*, *RUNX2*, *ALPL*), myeloid cells (*LYZ*, *FCGR3A*, *S100A9*, *APOE*, *CD14*), proliferative cells (*PCNA*, *TOP2A*, *CDK1*, *MKI67*), T/NK cells (*GZMK*, *GZMA*, *CD3D*, *TRAC*, *NKG7*), pericytes (*MYL9*, *TAGLN*, *RGS5*, *ACTA2*), osteoclasts (*CTSK*, *ACP5, MMP9*), endothelial cells (*CLDN5*, *CD34*, *CDH5*, *VWF*, *EGFL7*, *PECAM1*) (Fig. 1C, D). Figure 1E showed the distribution of each cell subpopulation across different patients. The proportion of cell type across different patients varied considerably, indicating that there was intertumoral heterogeneity in osteosarcoma.

### Single-cell RNA data reveals the compositional differences in ECs

**Fig. 2:**
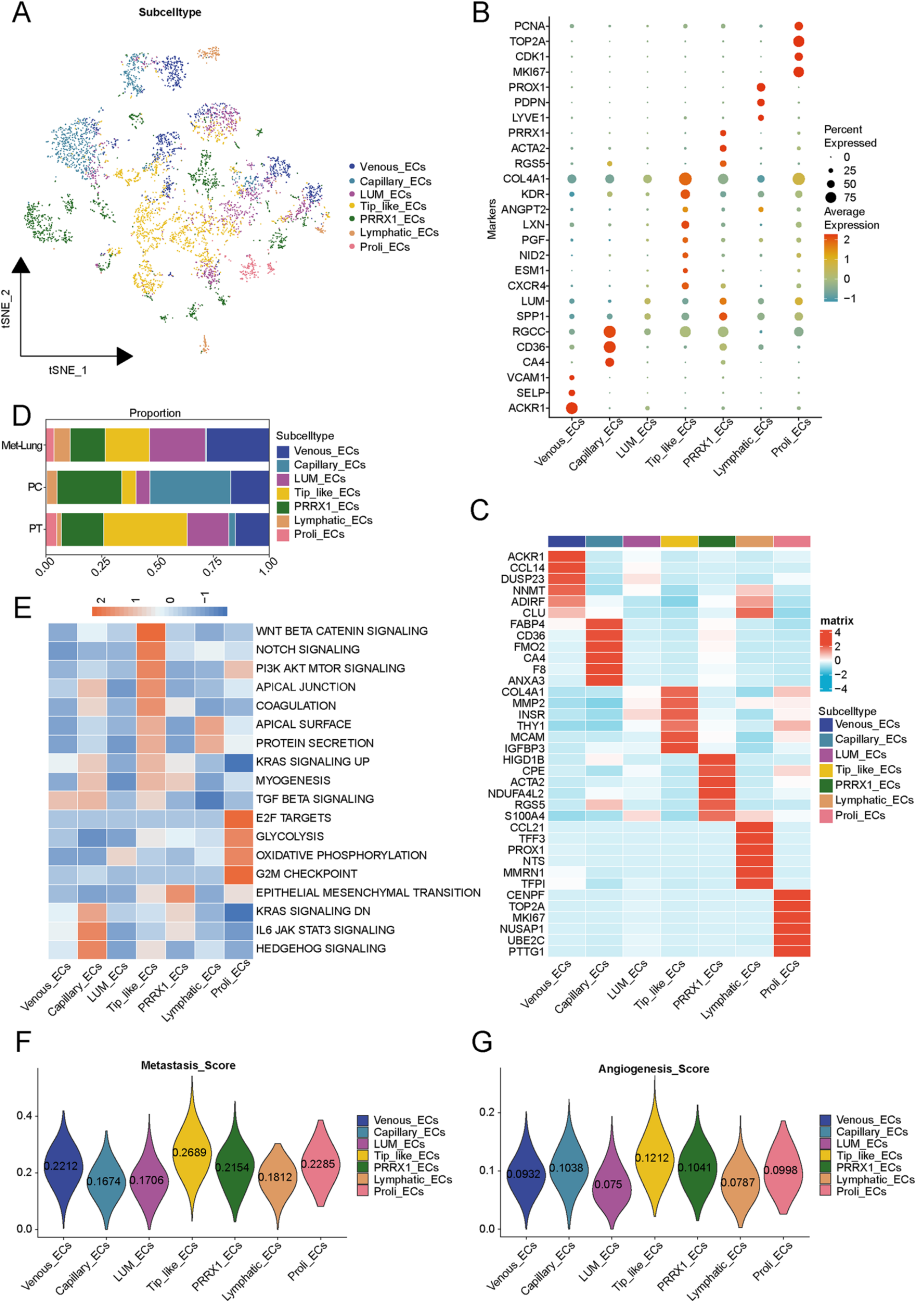
Classification of EC subtypes. **A** ECs were categorized into seven distinct subtypes. **B** The marker genes of each EC subtype. **C** Gene expression heatmap in EC subtypes. **D** Composition of EC subtypes among different groups. **E** GSVA analysis showed that the tip-like ECs subtype was significantly enriched in the NOTCH SIGNALING pathway, WNT BETA CATENIN SIGNALING, and APICAL JUNCTION pathway etc. **F** Lung metastasis scores were the highest in tip-like ECs by AUCell. **G** Angiogenesis scores were the highest in tip-like ECs by AUCell.(Proli: proliferative).

The ECs were extracted from the osteosarcoma cell atlas and re-clustered to construct an osteosarcoma ECs atlas (Fig. 2A). In this atlas, ECs were classified into seven subclusters: venous ECs (*ACKR1*, *SELP*, *VCAM1*), capillary ECs (*CA4*, *CD36*, *RGCC*), *LUM*_ECs (*SPP1*, *LUM*), tip-like ECs (*CXCR4*, *ESM1*, *NID2*, *PGF*, *LXN*, *ANGPT2, KDR, COL4A1*), *PRRX1*_ECs(*RGS5*, *ACTA2*, *PRRX1*), lymphatic ECs (*LYVE1*, *PDPN*, *PROX1*), proliferative ECs (*MKI67*, *CDK1*, *TOP2A*, *PCNA*) (Fig. 2B). Figure 2C showed the top 6 differentially expressed genes for each endothelial cell subpopulation.

### Tip-like ECs may promote angiogenesis and lung metastasis

Tip-like ECs were most abundant in the PT group and had a relatively higher abundance in the Met-Lung group compared to the PC group (Fig. 2D), suggesting their potential involvement in the oncogenesis and metastasis of osteosarcoma. Supplementary Fig. 1A showed the differentially expressed genes and pathways of tip-like ECs in different groups, which may be involved in the functional execution processes of tip-like ECs.

GSVA was used to explore the functional differences among different endothelial cell subpopulations, and the results indicated that tip-like ECs were primarily enriched in pathways related to cell proliferation and differentiation, including WNT BETA CATENIN SIGNALING, NOTCH SIGNALING, and PI3K AKT MTOR SIGNALING (Fig. 2E). AUCell scoring was performed on different ECs subpopulations based on metastasis and angiogenesis gene sets. The results revealed that tip-like ECs had the highest metastasis and angiogenesis scores (Fig. 2F, G), further supporting the notion that ECs might be closely associated with metastasis and angiogenesis of osteosarcoma. In summary, tip-like ECs may be involved in both tumor angiogenesis and metastasis. Thus, tip-like ECs were identified as the target cell cluster in this study.

### Analysis of cellular communication between tip-like ECs and osteoblasts

**Fig. 3:**
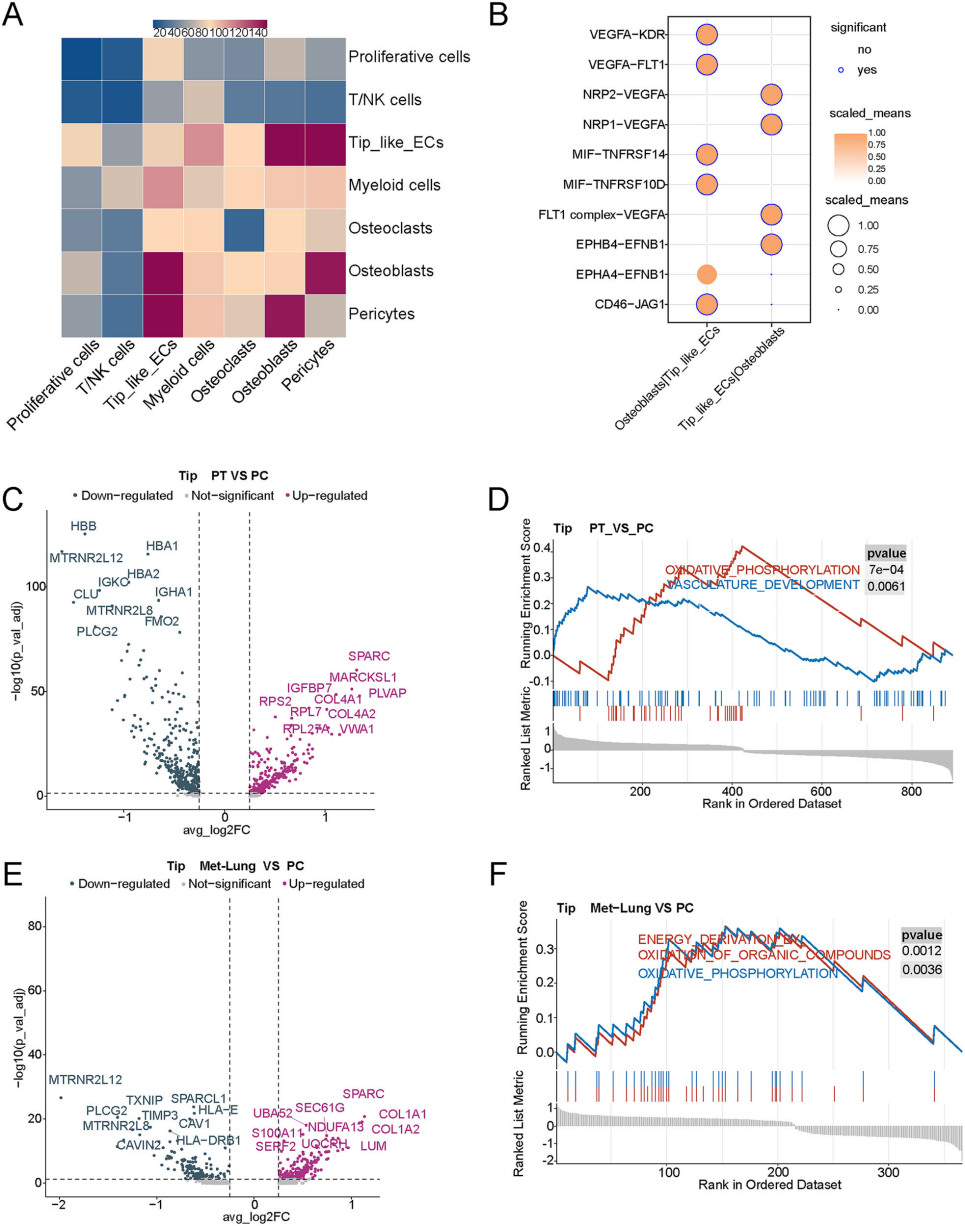
Cellular communication and differential gene analysis between osteosarcoma groups. **A** Heatmap of cellular interactions between tip-like ECs and other clusters. **B** Cellular ligand-receptor bubble maps of osteoblasts and tip-like ECs. **C** Differential gene volcano plots of tip-like ECs in the PT group versus the PC group. **D** GSEA plot of differentially expressed genes in tip-like ECs in PT versus the PC group. **E** Differential gene volcano plot of tip-like ECs in Met-Lung group versus the PC group. **F** GSEA plot of differentially expressed genes in tip-like ECs in Met_Lung versus the PC group (PT primary tumor, PC paracancerous, Met-Lung lung metastasis).

CellPhoneDB results indicated a large number of ligand-receptor pairs between tip-like ECs and osteoblasts, suggesting significant cellular interactions between these two cell types (Fig. 3A). Figure 3B showed the potential ligand-receptor pairs between tip-like ECs and osteoblasts.

### Differential expression and pathways of tip-like ECs across different groups

Compared to the PC group, tip-like ECs in PT exhibited higher expression of genes associated with cell migration, invasion, and angiogenesis, such as *MARCKSL1*, *PLVAP*, and *SPARC*^15–17^ (Fig. 3C). GSEA demonstrated significant enrichment of pathways, including VASCULATURE DEVELOPMENT and OXIDATIVE PHOSPHORYLATION, in tip-like ECs from the PT group (Fig. 3D). These results indicated that tip-like ECs in the PT group were more actively involved in angiogenesis. Compared to the PC group, tip-like ECs exhibited higher expression of genes associated with epithelial-mesenchymal transition (EMT), metastasis, and invasion, such as *SPARC*, *SEC61G* and *S100A11*^15,18,19^ (Fig. 3E). GSEA revealed significant enrichment of pathways, including ENERGY DERIVATION BY OXIDATION OF ORGANIC COMPOUNDS and OXIDATIVE PHOSPHORYLATION, potentially suggesting that the metabolism of tip-like ECs in the metastatic focus was highly active (Fig. 3F). To sum up, these results suggested that tip-like ECs exerted their potential pro-tumorigenic role by angiogenesis.

### The mutual interaction between tip-like ECs and chemotherapy

**Fig. 4:**
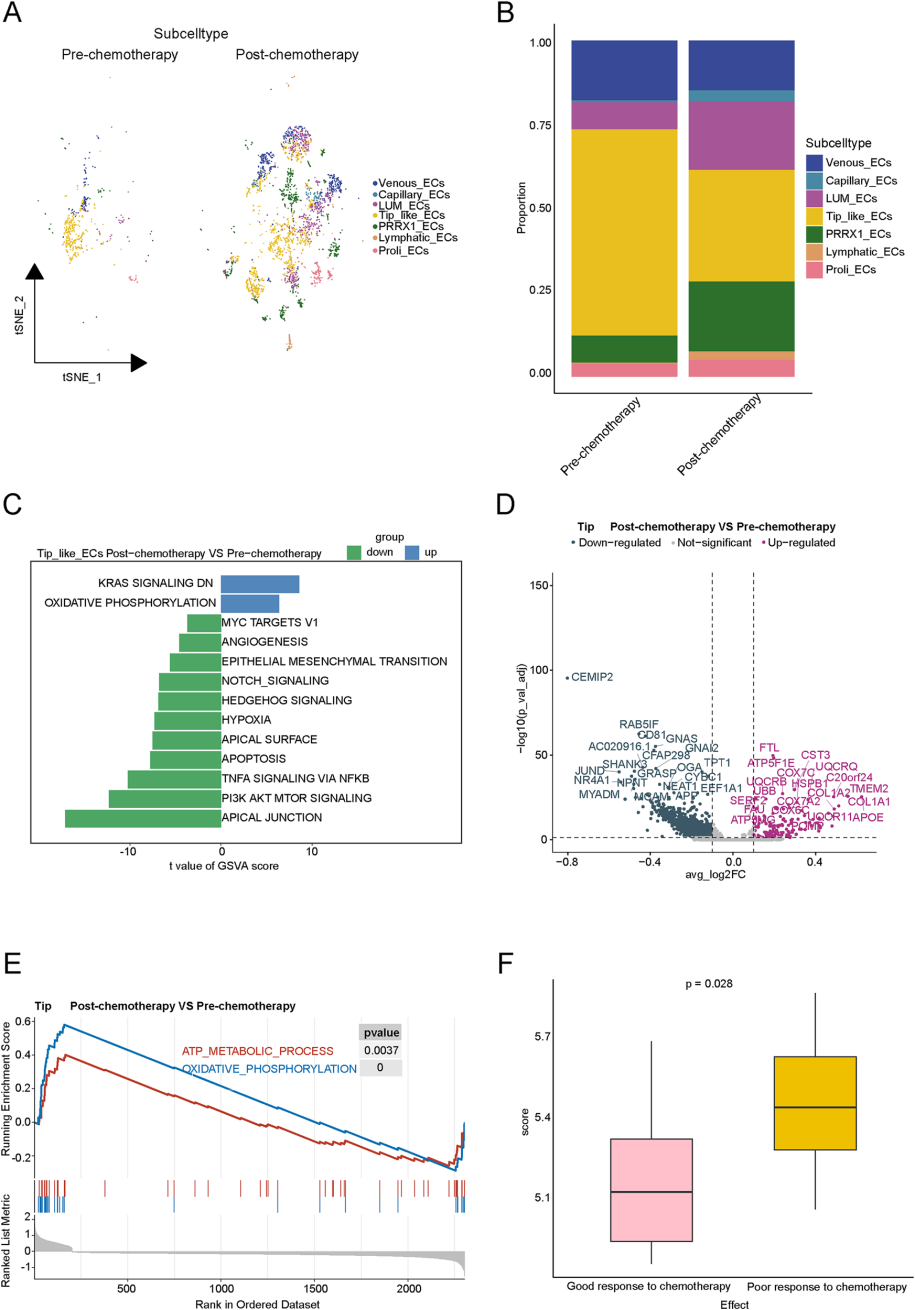
Landscape of EC subtypes in pre- and post-chemotherapy. **A** tSNE plots of ECs in different groups. **B** Composition of EC subtypes across different groups. **C** Differences in pathway activities scored per cell by GSVA between pre-chemotherapy and post-chemotherapy tip-like ECs. **D** Differential gene volcano plot of tip-like ECs between pre- and post-chemotherapy. **E** GSEA plot of differentially expressed genes in tip-like ECs between pre- and post-chemotherapy. **F** Scores of tip-like ECs in chemotherapy efficacy groups.

To investigate the role of tip-like ECs in osteosarcoma chemotherapy, the PT group samples were divided into pre- and post-chemotherapy subgroups (Fig. 4A). The proportion of tip-like ECs significantly decreased in post-chemotherapy group (Fig. 4B). The pathways related to angiogenesis, tumor invasion, and metastasis, including HEDGEHOG SIGNALING, ANGIOGENESIS, and EPITHELIAL-MESENCHYMAL TRANSITION, were downregulated in the post-chemotherapy group. These results implied the angiogenic capacity and pro-tumorigenic role of tip-like ECs might be diminished by chemotherapy (Fig. 4C).

In addition, *FTL* and *TMEM2*, genes associated with chemoresistance^20,21^, were upregulated in the post-chemotherapy group (Fig. 4D). Pathways such as ATP METABOLIC PROCESS and OXIDATIVE PHOSPHORYLATION were also significantly enriched, likely reflecting cellular responses to chemotherapy-induced stress (Fig. 4E). Patients in the TARGET dataset were scored based on the top 30 genes expressed in tip-like ECs. The result showed that patients with poor chemotherapy responses had higher scores, suggesting that tip-like ECs may contribute to chemoresistance (Fig. 4F). Altogether, these results suggested that tip-like ECs might be involved in osteosarcoma chemotherapy resistance.

### Pseudotemporal trajectory analysis of EC subtypes in osteosarcoma

**Fig. 5:**
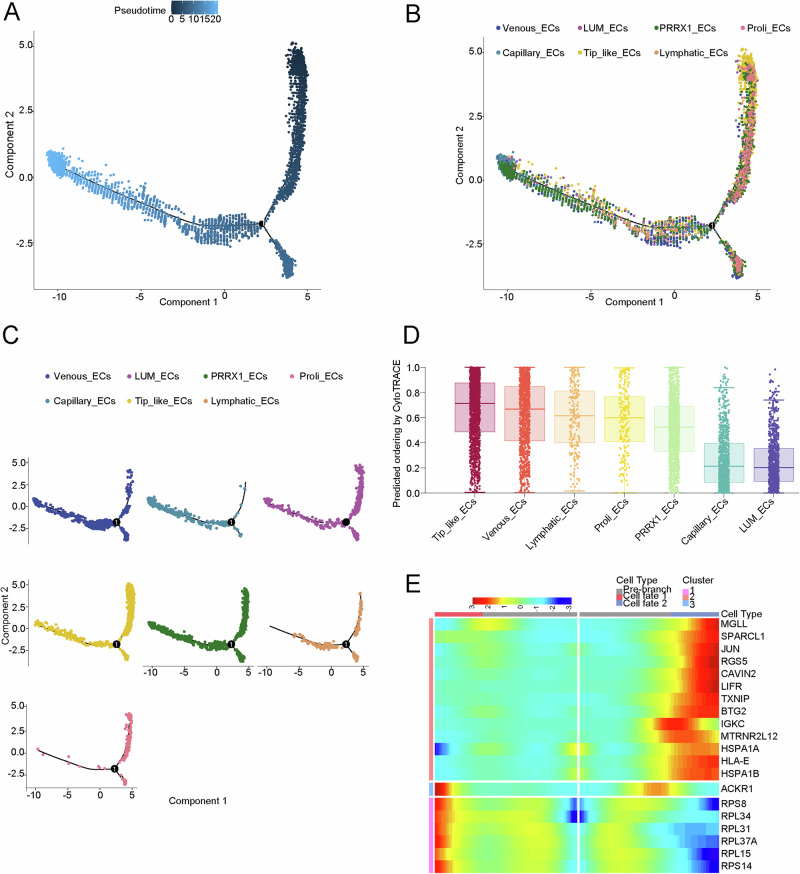
Pseudotemporal trajectory analysis of EC subtypes. **A**–**C** Pseudotemporal analysis of EC subtypes. **D** CytoTRACE plot of EC subtypes. **E** Heatmap depicts the expression of the top 20 genes in EC subtypes across two branches, classified into three gene clusters.

To dissect the developmental trajectory of the EC lineage in osteosarcoma and identify dynamic changes in cellular status, we performed pseudo-temporal cell trajectory analysis and generated a three-branch trajectory (Fig. 5A). Tip-like ECs marked the beginning of differentiation, while *LUM*_ECs represented the endpoint (Fig. 5A–C). CytoTRACE result showed that tip-like ECs had the highest stemness score further identifying the tip-like ECs as the starting point of EC clusters (Fig. 5D). The heatmap displayed genes during differentiation from the initial state to cell fate 1 or 2, which may be involved in the differentiation of tip-like ECs (Fig. 5E).

### *MCAM* plays a key role in tip-like ECs

**Fig. 6:**
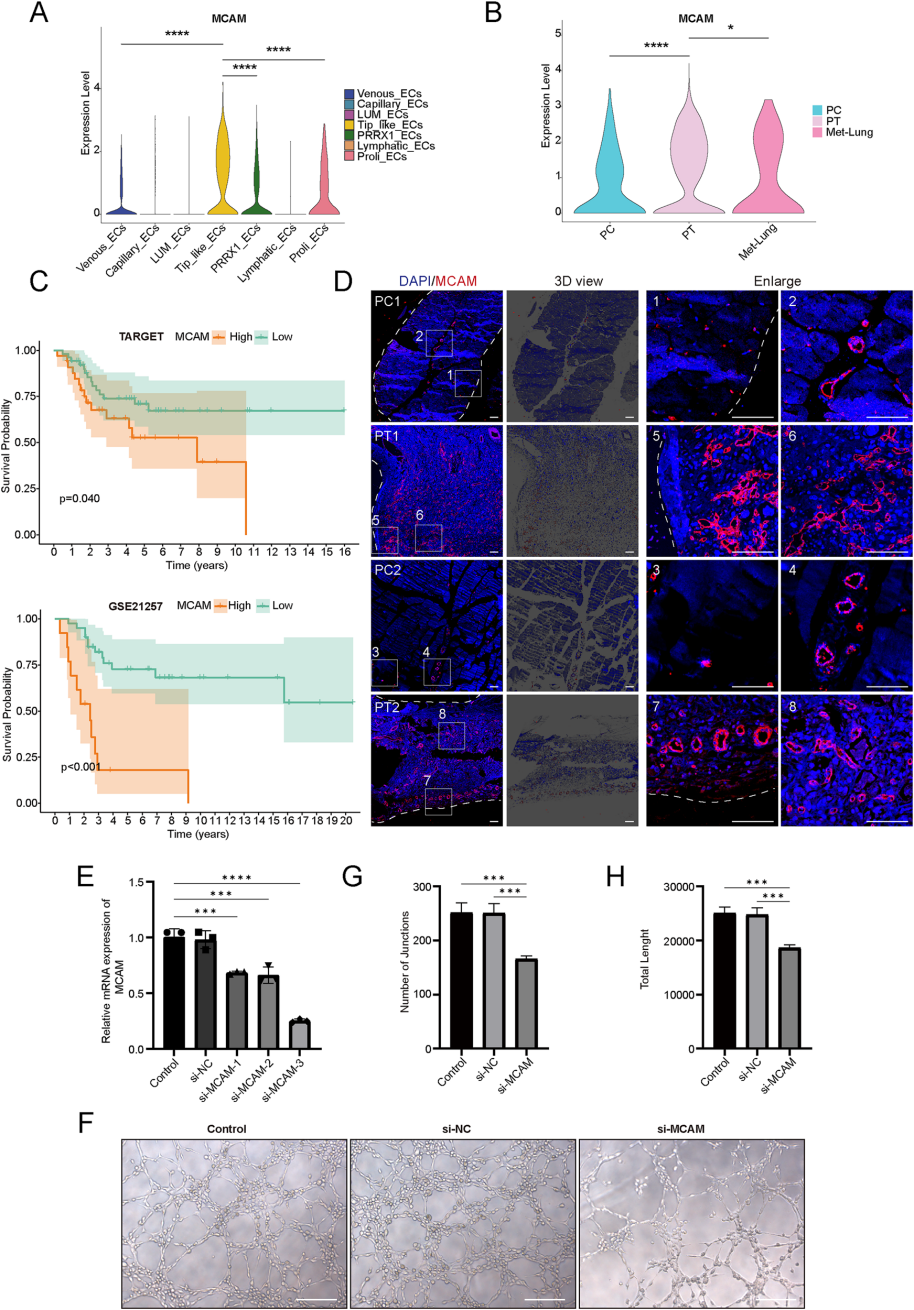
Validation of the effect of *MCAM* on osteosarcoma progression. **A** Violin plot of *MCAM* in EC subtypes. **B** Violin plot of the expression of *MCAM* in different sample groups. **C** Prognostic analysis of patients with high and low *MCAM* expression. **D** Representative immunofluorescence images of *MCAM* in osteosarcoma tissue and peritumoral tissue (*n* = 2). **E** PCR detection of siRNA-*MCAM* knockdown efficiency. **F** Representative images of tube formation assay. **G** Number of connections of cell-forming tubes. **H** Total length of cell-forming tubes. (NC negative control, PC paracancerous, PT primary tumor, Scale bar = 200 μm,***p < 0.001, ****p < 0.0001).

To identify key functional genes, we calculated the differentially expressed genes in tip-like ECs. The results showed that melanoma cell adhesion molecule (*MCAM*) was highly expressed in tip-like ECs compared to other EC subpopulations (Fig. 6A). Moreover, the expression of *MCAM* was high in tip-like ECs within the PT group (Fig. 6B). *MCAM*, also known as *CD146*, is associated with angiogenesis, metastasis, and chemoresistance^22–24^. Pseudotime analysis showed that *MCAM* was mainly expressed in tip-like ECs and gradually decreased during EC development, suggesting that *MCAM* may be involved in the differentiation of tip-like ECs (Supplementary Fig. 2A, B). Survival analysis based on the TARGET and GSE21257 datasets was conducted, and the result indicated that patients with high *MCAM* expression had a poorer prognosis (Fig. 6C). To validate the impact of *MCAM* on vasculature at the tissue level, we performed immunofluorescence staining. The result showed that *MCAM* expression around blood vessels in osteosarcoma tissue (PT) was significantly higher compared to the peritumoral tissue (PC) group (Fig. 6D). In summary, the above results suggested that *MCAM* could serve as a hub gene for tip-like ECs.

### Knockdown of *MCAM* inhibits angiogenesis in vitro

The key role of *MCAM* was further confirmed via in vitro experiments. PCR was used to assess the silencing efficiency of the si-*MCAM* plasmid, and si-*MCAM*-3 was found to have the highest silencing efficiency. Therefore, si-*MCAM*-3 was selected for subsequent experiments (Fig. 6E). Matrigel tube formation assay revealed that si-*MCAM* could significantly suppress the angiogenic capability of HUVECs (Fig. 6F–H).

### scRNA-seq combined with spatial transcriptomics reveals *MCAM*-mediated spatial characterization of ECs

**Fig. 7:**
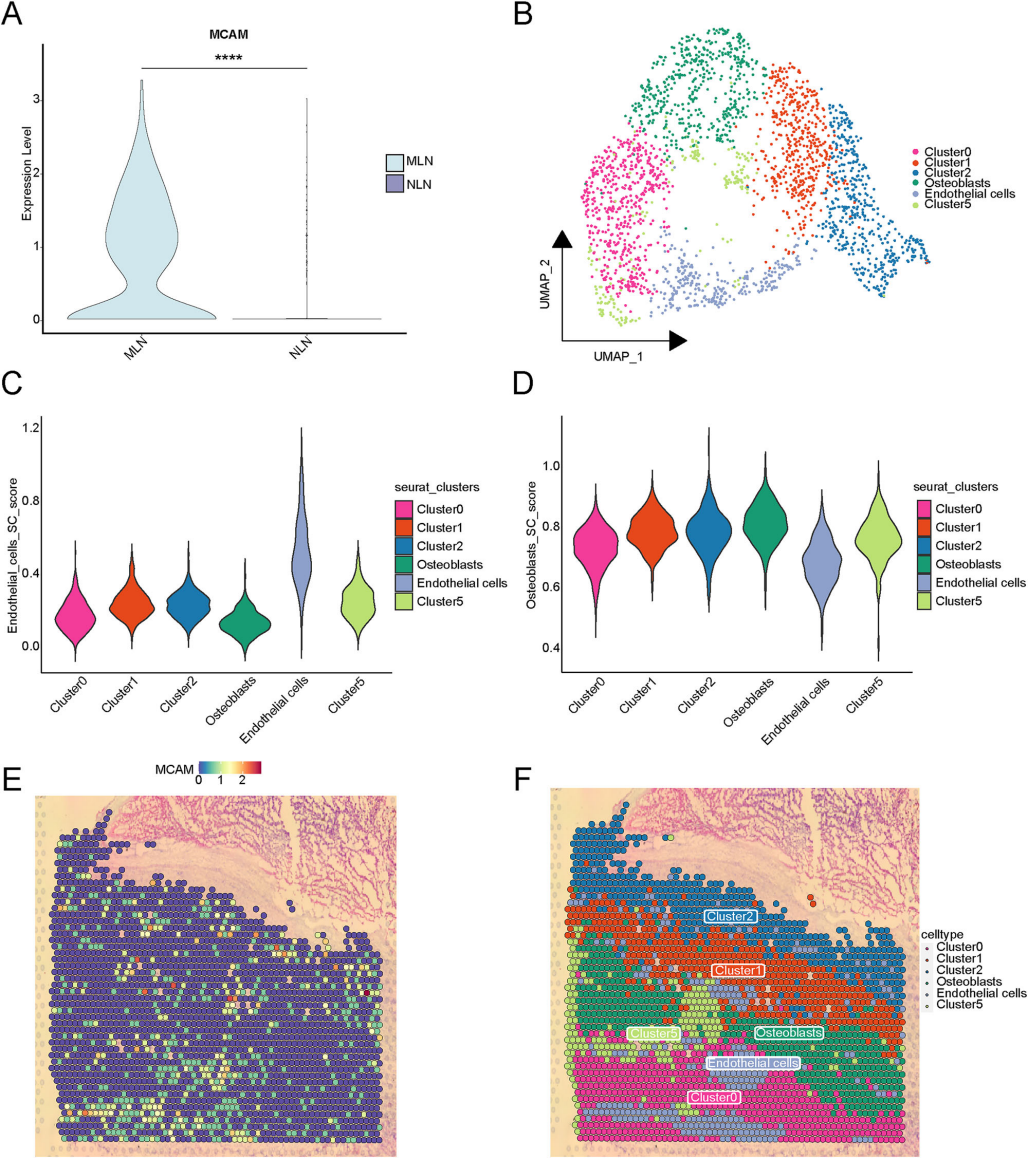
Single-cell and spatial transcriptomic characterization of ECs in metastatic lymph node samples. **A** Violin plot of *MCAM* expression in MLN and NLN groups. **B** UMAP plot of ST data from MLN. **C** AddModuleScore analysis of ECs gene set for ST cell clusters. **D** AddModuleScore analysis of osteoblasts gene set for ST cell clusters. **E** Spatial location of *MCAM* expression. **F** Spatial location of cell clusters in MLN (ST spatial transcriptomics, MLN metastatic lymph node, NLN normal lymph node).

To investigate whether *MCAM* influences lymph node metastasis in osteosarcoma, we analyzed its expression in normal (NLNs) and metastatic lymph nodes (MLNs) using scRNA-seq data. *MCAM* was found to be more highly expressed in ECs of MLNs (Fig. 7A). To further explore the spatial relationship between ECs and osteoblasts, spatial transcriptomics (ST) data from a metastatic lymph node sample was analyzed. After dimensionality reduction and clustering, all spots were grouped into six clusters (Fig. 7B). Using the AddModuleScore algorithm, cluster 4 was identified as endothelial cells (Fig. 7C), and cluster 3 was identified as osteoblasts (or tumor cells) (Fig. 7D). In the ST section, *MCAM* expression was co-localized with endothelial cells, confirming the presence of *MCAM*^+^ ECs (Fig. 7E, F). Moreover, *MCAM*^+^ ECs were found adjacent to osteoblasts (Fig. 7E, F), suggesting potential cell-cell interactions between *MCAM*^+^ ECs and tumor cells.

### Drug sensitivity analysis of *MCAM* gene

To investigate the sensitivity of the target gene to various anticancer drugs, samples were grouped based on *MCAM* gene expression, and the association between *MCAM* levels and drug sensitivity was analyzed. The results indicated that patients with high *MCAM* expression exhibited greater sensitivity to AICAR, Axitinib, Bosutinib, CEP-701, Methotrexate, and Sorafenib (all *P* < 0.05, Supplementary Fig. 3A). Conversely, patients in the low expression group showed greater sensitivity to TGX221 and Gefitinib. Additionally, the association between the risk score and sensitivity to chemotherapy drugs was confirmed (Supplementary Fig. 3B).

## Discussion

Osteosarcoma, a highly invasive malignant tumor, significantly reduces patient survival rates once metastasis occurs^25^. ECs play a pivotal role in promoting tumor growth, metastasis, and immune evasion^26,27^. As a subtype of ECs, tip-like ECs guide directional vascular growth, supplying essential nutrients to tumors and playing a crucial role in modulating the tumor microenvironment^28,29^. However, despite the widely acknowledged importance of tip-like ECs in angiogenesis and tumor development, research on their role in osteosarcoma remains limited. In this study, a single-cell atlas of osteosarcoma ECs was constructed by integrating scRNA-seq data. A novel subtype of tip-like ECs, potentially associated with osteosarcoma metastasis, was identified. Through further experiments, the role of *MCAM* in promoting angiogenesis was validated. Besides, the presence of *MCAM*^**+**^ ECs in metastatic lymph nodes (MLNs) was confirmed by scRNA sequencing and spatial transcriptomics, and these cells were found to be closely associated with tumor cells.

Endothelial cells (ECs) are important stromal cells within the tumor microenvironment, playing a crucial role in tumorigenesis and metastasis^12^. Tip-like ECs, a subpopulation of endothelial cells, play a critical role in angiogenesis^30^. Zhang et al.^29^ observed that tip-like ECs were predominantly found in tumor tissues. These cells not only promoted tumor angiogenesis but also inhibited anti-tumor immune responses. Another study found that regulators of angiogenesis were overexpressed in tip-like ECs within tumors, indicating that tip-like ECs play a significant role in promoting angiogenesis and tumor progression^31^. In our study, scRNA-seq was performed on osteosarcoma samples to construct an ECs atlas. The ECs were categorized into seven subtypes, with a novel subtype of tip-like ECs being identified. Our data suggest that tip-like ECs may be associated with angiogenesis and metastasis in osteosarcoma, which is consistent with the aforementioned previous reports.

Melanoma Cell Adhesion Molecule (*MCAM*, also known as *CD146*), originally identified as an endothelial marker linked to angiogenesis during tumor progression, is widely expressed throughout the vascular system^32,33^. *MCAM* plays a crucial role in processes such as angiogenesis, cell migration, and signal transduction^34^. Additionally, *MCAM* is critically involved in tumor aggressiveness, metastasis, and poor prognosis^22,35^. For example, in breast cancer, *MCAM* has been shown to promote tumor dissemination and metastasis by participating in the epithelial-mesenchymal transition process^36^. In melanoma, the interaction between *MCAM* and Galectin-3 promotes tumor progression by activating the AKT signaling pathway^37^. In our study, *MCAM* was also identified as a key gene associated with angiogenesis and metastasis in tip-like ECs, making it a valuable therapeutic target in osteosarcoma treatment.

In conclusion, a population of tip-like ECs was identified in osteosarcoma, contributing to angiogenesis and metastasis. *MCAM* was identified as a functional gene for tip-like ECs and could be used as a therapeutic target for blocking metastasis.

However, our study presented several limitations. First, the spatial transcriptomics data included in this study were limited, and more spatial transcriptomics data from primary tumors and metastatic lesions are necessary. Second, most of the findings in this study are based on transcriptomics, and multi-omics data would provide more robust support for the conclusions. Third, the functional validation of *MCAM* in this study remains superficial, and we plan to conduct more in-depth mechanistic and in vivo experiments in future research.

## Methods

### Ethics statement

All patients provided informed consent, and the study was approved by the Ethics Committee of the First Affiliated Hospital of Guangxi Medical University (Ethics number:2019KY-E-097). The study was performed in accordance with the Declaration of Helsinki.

### Data sources

In this study, a total of 25 scRNA-seq samples were included, 18 of which were obtained from our previous research^38^. Detailed method descriptions are provided in Supplementary File 4. The surgically resected samples were collected, cut into 1 mm^3^ pieces and digested into cell suspensions for further processing. Following single-cell isolation, single-cell transcriptome amplification and library preparation were performed according to 10X Genomics. The raw FASTQ files were then aligned to the human genome reference (GRCh38) using Cell Ranger software (v4.0.0). This process generated the barcode.tsv, feature.tsv, and matrix.mtx files. We also retrieved single-cell sequencing data from seven osteosarcoma (Met-Lung1, Met-Lung2, PT09-13) cases available in public databases (GSE152048). Additionally, spatial transcriptomic data of a metastatic lymph node, sourced from our previous study, was included. Bulk sequencing data and clinical information of osteosarcoma patients were obtained from the TARGET database (https://ocg.cancer.gov/programs/target) and GEO database (GSE21257).

### Quality control for scRNA-seq data

The scRNA-seq data were analyzed using the Seurat package (v4.3.0.1). Preliminary filtering criteria for genes and cells were set at min.cells = 3 and min.features = 200. Quality control thresholds were applied as follows: nFeature_RNA > 500, nFeature_RNA < 4500, and percent.mt < 10.

### Dimensionality reduction and clustering

The scRNA-seq data were normalized using the *NormalizeData* function in the Seurat package. The top 2000 highly variable genes were identified from normalized scRNA-seq data using the *FindVariableFeatures* function. The expression matrix was then scaled using the *ScaleData* function. After PCA, the Harmony (v1.2.0) package was applied to integrate the data and remove batch effects, with the top 20 principal components (PCs) selected to ensure the accuracy and reliability. Cluster analysis was performed using the *FindNeighbors* and *FindClusters* functions. The results were visualized through t-distributed stochastic neighbor embedding (t-SNE) with a resolution of 0.03. Subsequently, endothelial cells were extracted and re-clustered (resolution = 0.03), identifying four distinct subtypes of ECs (cluster0-cluster3). Cluster0 was further subdivided at a resolution of 0.1. Ultimately, seven EC subtypes were obtained and visualized.

### Differentially expressed genes identification and functional enrichment analysis

DEGs of ECs between different sample groups were identified using the *FindMarkers* function. A volcano plot was generated to visualize DEGs. Gene Set Variation Analysis (GSVA) (v1.42.0) was utilized to calculate the pathway score. Gene Set Enrichment Analysis (GSEA) (v1.2) was performed to detect biological functions significantly enriched in specified EC subtype.

### Constructing single-cell pseudotime differentiation trajectory

To investigate the evolutionary and differentiation processes among ECs subtypes, the Monocle2 (v2.22.0) package was used. Trajectory analysis was performed using genes with an average expression ≥ 0.1. Dimensionality reduction was achieved using the DDRTree method, followed by sorting and visualization of cells via the *plot_cell_trajectory* function. The cellular trajectory was then ordered based on pseudotime. A pseudotime heatmap was generated to demonstrate the gene expression over time. Additionally, pseudotime analysis was performed for the *MCAM* gene.

### CytoTRACE evaluation of cell stemness and differentiation potential

CytoTRACE (v0.3.3) is an algorithm used to predict cellular differentiation status from scRNA-seq data. Higher CytoTRACE scores indicate less differentiation and greater pluripotency. CytoTRACE can assist trajectory algorithms (such as Monocle) in defining the starting point of cell development.

### Scoring based on relevant marker genes

The AUCell algorithm was employed to calculate the enrichment scores of genes associated with angiogenesis and metastasis. Angiogenesis-related gene sets were obtained from MSigDB (https://www.gsea-msigdb.org/gsea/msigdb/index.jsp), and genes positively associated with lung metastasis were gathered from relevant literature. Additionally, the “*AddModuleScore*” function was applied to assess gene set scores.

### ssGSEA

Chemotherapy response data of 22 samples were extracted from the TARGET database (https://ocg.cancer.gov/programs/target/) for single-sample Gene Set Enrichment Analysis (ssGSEA). First, the top 30 highly expressed genes of tip-like ECs were extracted, and the ssGSEA algorithm was used to score these patients. Subsequently, based on the histologic response scores, patients were divided into two groups: a poor response to chemotherapy with scores between 0–90, and a good response to chemotherapy with scores between 91–100. Finally, the t-test was performed to compare the ssGSEA scores between the two groups of patients, in order to determine whether tip-like ECs were associated with chemotherapy.

### Analysis of intercellular interactions

To investigate intercellular communication networks, CellPhoneDB (v3.0.0) was used to analyze the interactions between ECs and other cell types. The results were visualized using heatmaps and bubble plots.

### Spatial transcriptomics data analysis

Reads were mapped to the human GRCh38 genome using SpaceRanger. Spots with fewer than 25% mitochondrial genes, more than 500 detected gene counts and more than 1500 total counts were retained. We applied the *SCTransform* and *RunPCA* functions for data normalization and principal component analysis. Unsupervised clustering methods were then used to cluster similar spatial transcriptomics (ST) spots. The top 100 differential genes for each cell cluster identified from the scRNA-seq data were used for ST data analysis. The *AddModuleScore* function was used to calculate the scores for each cluster in the ST data, which were then used to annotate the cell types of the ST clusters.

### ClusterGVis

The specific genes of the clusters were identified using the *FindAllMarkers* function. Functional enrichment analysis was performed using the ClusterGVis (v0.1.1) R package (https://github.com/junjunlab/ClusterGVis).

### Survival analysis

Bulk RNA sequencing data from 85 osteosarcoma patients in the TARGET database and 53 patients in the GEO database (GSE21257) were used. The *surv_cutpoint* function was used to determine the cutoff value for *MCAM*, after which patients were divided into high and low expression groups. Survival differences were conducted with the R packages of survival (v3.5-5) and survminer (v0.4.9).

### *MCAM* gene-related drug sensitivity

The pRRophetic (v0.5) package was used to calculate the half-maximal inhibitory concentration (IC50) for each drug. Samples from the TARGET database were divided into high-risk and low-risk groups based on the median expression level of *MCAM*. The *pRRopheticPredict* function was used to predict the drug sensitivity scores for each patient, and a Wilcoxon test was performed to compare the differences in scores between the two groups of patients. Spearman correlation analysis was performed to evaluate the relationship between risk scores and IC50 values. Lower IC50 values indicated higher drug sensitivity.

### Cell cultures and siRNA transfection

Human umbilical vein endothelial cells (HUVECs) were purchased from the China Center for Type Culture Collection (Wuhan, China). The cells were cultured in RPMI-1640 medium (Gibco, USA) supplemented with 10% fetal bovine serum (Sijiqing, China) and 1% penicillin-streptomycin mixture (Solarbio, China), and maintained in a cell culture incubator at 37 °C with 5% CO_2_. For siRNA transfection, HUVECs were seeded in 6-well plates, and interfered with the transfection complex formed by RNATransMate with siRNA-*MCAM* or siRNA-NC (BioWorks, Shanghai, China) for 48 h. The knockdown efficiency of the siRNA was assessed by PCR. Subsequently, the cells were used for tube formation assays.

### qRT-PCR

The expression level of *MCAM* mRNA in HUVECs after siRNA intervention was detected by qRT-PCR. The cDNA was obtained by reverse transcription of mRNA with PrimeScript RT Master Mix Reverse Transcription Kit (TaKaRa, Japan). GAPDH was used as an internal reference and the expression levels of *MCAM* in the various cell groups were measured using SYBR Green I real-time fluorescence quantitative PCR. The primer sequences were: *MCAM* F:5’-TGTTGGAGACAGGTGTTGAATGC-3’, R: 5’-GTTGTGTTGGAGTCTGGTGTGAG-3’; GAPDH F:5’-CAGGAGGCATTGCTGATGAT-3’, R: 5’-GAAGGCTGGGGCTCATTT-3’. The qRT-PCR was designed according to the FastStart Universal SYBR Green Master instructions (Roche, Switzerland) for setting up the reaction program and reaction system, and the relative quantitative analysis was performed by the 2^-∆∆Ct^ method.

### Tube formation assay

HUVECs were divided into a control group, an siRNA-NC transfected group and an siRNA-*MCAM* transfected group. Subsequently, the cells were seeded into 96-well plates that was coated with matrigel (50 μL/well) at a density of 3 × 10^4^ cells per well. After incubating for 3 hours in an incubator set at 37 °C with 5% CO_2_, photographs were taken with a light microscope. The number of connections and total length of cell-forming tubes were analyzed and quantified using Image J software.

### Immunofluorescence staining of formalin‐fixed, paraffin‐embedded tissue

Tissue sections and immunofluorescence staining of primary tumor (PT) and paracancerous (PC) specimens were performed according to standard protocols. All sections of formalin-fixed paraffin-embedded (FFPE) were cut to a thickness of 3 μm and underwent deparaffinization and rehydration using a gradient of dewaxing solution and ethanol. Antigen retrieval was performed by incubating the sections in immunohistochemical antigen retrieval buffer (EDTA method) under high pressure and heat. After a 15-minute soaking in 3% hydrogen peroxide, the sections were blocked with blocking solution. The sections were then incubated overnight at 4 °C with the primary antibody. Subsequently, the secondary antibody incubation was performed for 1 hour at room temperature under light-protected conditions. After staining with DAPI for 10 minutes, the sections were covered with coverslips for observation. The following antibodies and reagents were used: *CD146* (1:100, Abcam, ab75769), blocking goat serum (1:10, Solarbio, SL038), Alexa Fluor ® 555 Conjugate (1:100, Cell Signaling Technology, 4413)

### Statistical analysis

In this study, data were analyzed and generated using R software (v4.0.5 and v4.1.0). *P* < 0.05 was the significance threshold. A nonparametric Wilcoxon rank-sum test was used for the functional gene and drug sensitivity scores. The Chi-square test was used to compare cell proportions between groups. An independent samples t-test was applied to compare ssGSEA scores between groups. Kaplan-Meier curves with log-rank statistics were used to assess overall survival.

## Supplementary information

Supplementary information

Supplementary File 4

## Data Availability

The in-house sequencing raw data for this study are stored in GSE162454 (https://www.ncbi.nlm.nih.gov/geo/query/acc.cgi?acc=GSE162454) and HRA007229 (https://ngdc.cncb.ac.cn/gsa-human/browse/). Public scRNA-seq datasets were retrieved from the Gene Expression Omnibus (GEO) database (access number: GSE152048). Bulk RNA-seq data of osteosarcoma were retrieved from GSE21257 and the TARGET database (https://ocg.cancer.gov/programs/target). Based on patient privacy considerations, the data are available under restricted conditions. Requests for access to the patient data in this study require a detailed research proposal, which must be submitted via email to the corresponding author for approval.

## Code availability

The code used for all processing and analysis is available at https://github.com/haijun2595/Endotheliocyte.
