# Supplementary material for: Single-cell and spatial transcriptomics reveals the key role of MCAM+ tip-like endothelial cells in osteosarcoma metastasis
Source: NPJ Precis Oncol. 2025 Apr 13;9:104. doi: 10.1038/s41698-025-00896-8 (PMC11993737; doi:10.1038/s41698-025-00896-8)
Supplement: Supplementary file 1 — Supplementary information [file 41698_2025_896_MOESM1_ESM.pdf]

Supplementary Figure 1

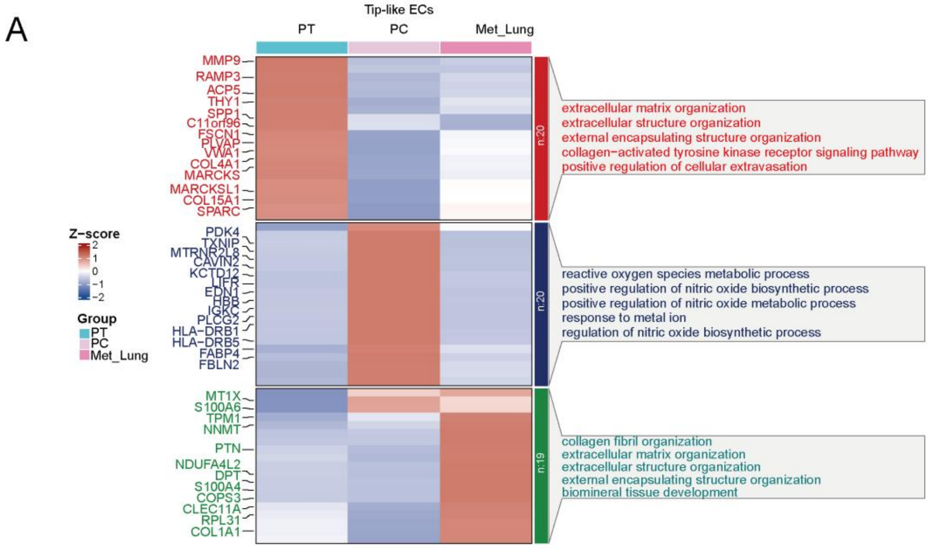

Supplementary Figure 1. ClusterGVis analysis of tip-like ECs across groups. (A) ClusterGVis showed the gene expression and functional pathways of tip-like ECs across different groups.

Supplementary Figure 2.

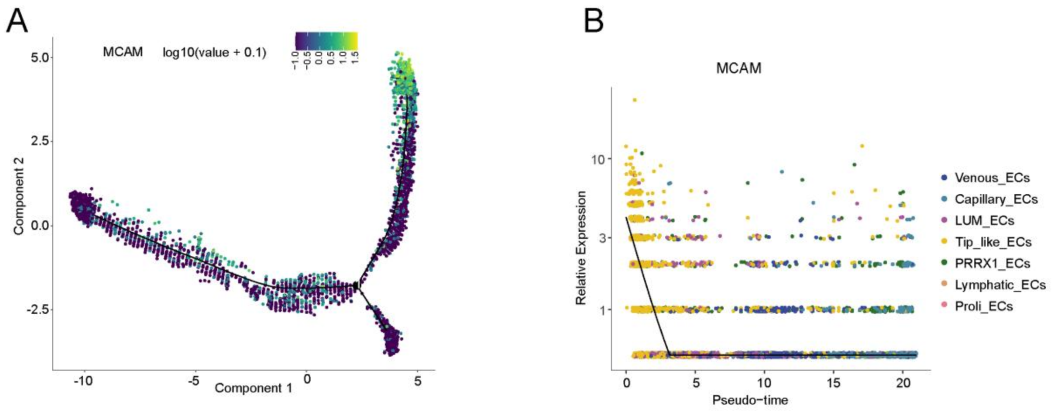

Supplementary Figure 2. *MCAM* expression in pseudotime analysis. (A) Trajectory plot showing the change in *MCAM* expression over differentiation time; (B) Changes in *MCAM* expression levels during the cell pseudotime differentiation process.

Supplementary Figure 3.

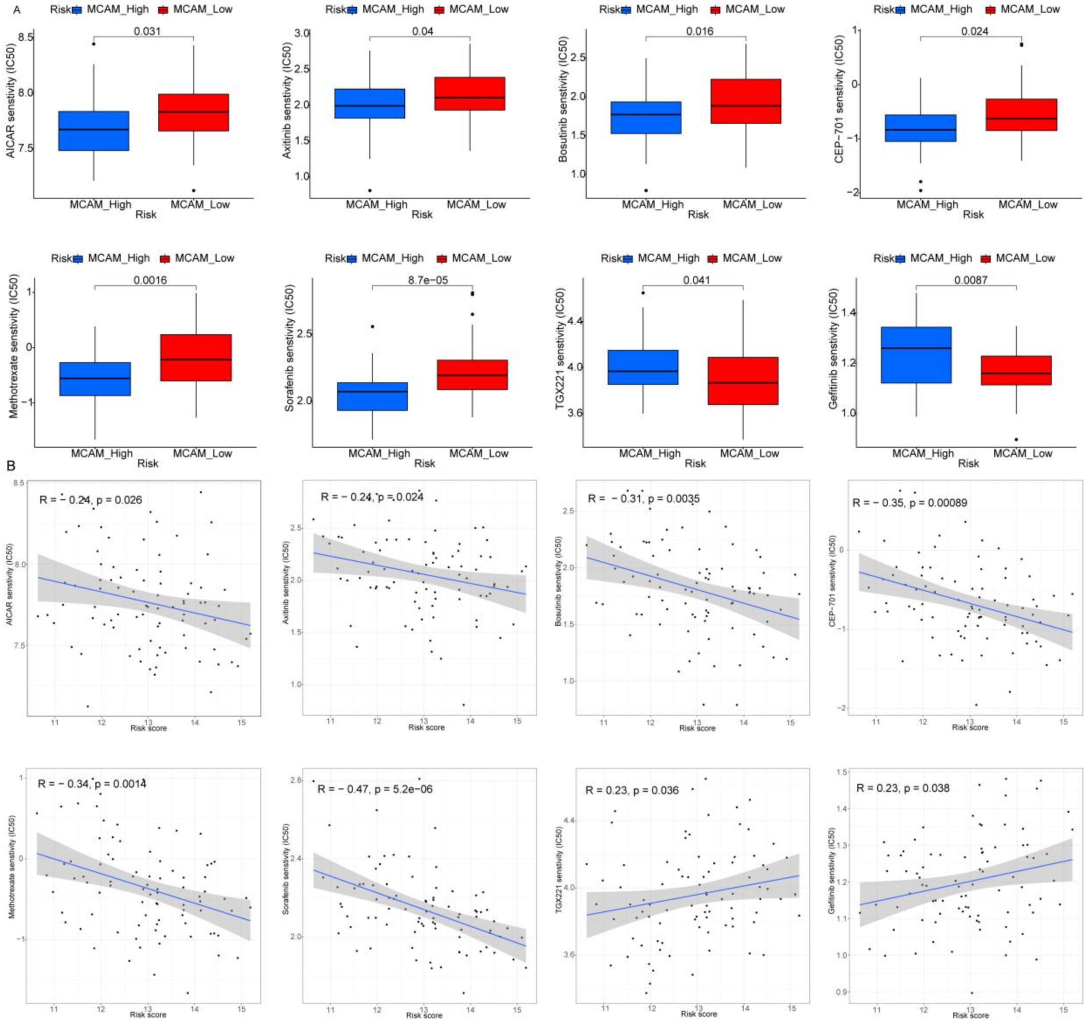

Supplementary Figure 3. Drug sensitivity analysis of the *MCAM* gene. (A) Differences in IC50 values of AICAR, Axitinib, Bosutinib, CEP-701, Methotrexate, Sorafenib, TGX221 and Gefitinib between *MCAM*_High and *MCAM*_Low groups. (B) Correlation analysis between risk score and drug IC50 values.
