# Supplementary material for: Single-cell and spatial transcriptomics reveals the key role of MCAM+ tip-like endothelial cells in osteosarcoma metastasis
Source: NPJ Precis Oncol. 2025 Apr 13;9:104. doi: 10.1038/s41698-025-00896-8 (PMC11993737; doi:10.1038/s41698-025-00896-8)
Supplement: Supplementary file 2 — Supplementary File 4 [file 41698_2025_896_MOESM2_ESM.pdf]

Supplementary File 4.

Collection of patient clinical samples

The inclusion and exclusion criteria for osteosarcoma samples were as follows: Inclusion criteria: 1) Histopathologically confirmed diagnosis of osteosarcoma following surgical resection; 2) Lesions localized to the extremities. Exclusion criteria: 1) Comorbid conditions such as fever, infection, or lymph node disorders; 2) Patients with recurrent osteosarcoma; 3) Patients lacking surgical or biopsy interventions, rendering sample acquisition impossible. Lymph node metastasis was diagnosed pathologically: metastatic lymph nodes exhibited complete effacement of normal nodal architecture with infiltration by pleomorphic tumor cells exhibiting enlarged, hyperchromatic nuclei and variable cell sizes; non-metastatic lymph nodes displayed preserved architecture with no evidence of tumor cells, displaying either normal lymphoid tissue or reactive hyperplasia.

Before sampling, patient medical histories and clinical data (including disease history, treatment regimens, and imaging findings) were comprehensively documented to enable correlation analysis between sequencing data and clinical data. Additionally, preoperative imaging studies were systematically reviewed to localize tumor-involved soft tissue regions, ensuring rapid intraoperative identification of viable osteosarcoma tissues. Following surgical resection, tumors were bisected along the longitudinal axis, and multi-directional sampling was performed in fish-flesh-like soft tissue regions. The tumor tissue was washed twice with PBS and then placed on ice for preservation. All procedures were conducted under aseptic conditions to eliminate microbial contamination risks for sequencing. Necrotic tissues and sclerotic bone were strictly excluded to mitigate risks of insufficient cell numbers or sequencing instrument obstruction. Reactive edematous soft tissue was similar to osteosarcoma tissue, and it was important to distinguish between them. The former was softer in texture, while osteosarcoma was more resilient and often accompanied by ossification. Post-rinsing inspection further distinguished inflammatory edematous tissue by its translucent appearance. The sample collection and processing procedure

was completed within 20 minutes to prevent RNA degradation or cellular viability loss.

Preparation of single-cell suspension

The specimens were washed twice with pre-chilled RPMI-1640 medium supplemented with 0.04% BSA. The tissue was minced into small pieces approximately 0.5 mm × 0.5 mm × 0.5 mm in size. Then, a freshly prepared enzymatic digestion solution was added to these pieces, and they were placed in a constant temperature incubator and underwent digestion at 37°C for 30 minutes, with gentle inversion every 5 minutes. The digested cell suspension was filtered through a 40 μm cell strainer and centrifuged at 300 × g for 5 minutes at 4°C. The resulting pellet was resuspended in an appropriate volume of medium, mixed with an equal volume of red blood cell lysis buffer, and allowed to stand at 4°C for 10 minutes. The cell suspension was then centrifuged at 300 × g for 5 minutes, and the supernatant was discarded. After washing, the pellet was resuspended in 100 μL of medium, and cell counting was performed using an automated cell counter.

Water-in-oil system, library construction, and sequencing

The water-in-oil system setup, library preparation, and high-throughput sequencing were performed using the 10x Genomics Chromium platform with technical support from OE Biotech Co., Ltd. (Shanghai, China). Detailed procedures were as follows: The water-in-oil single-cell micro-reaction system (Gel Bead-in-Emulsions, GEMs) was constructed using the 10x Genomics Chromium system, through which RNA molecules were transcribed into cDNA with unique barcodes. Subsequently, the GEMs were subjected to oil phase disruption and purification, and the quantity of target cDNAs was amplified via PCR to reach the level required for library construction. Once the library construction was finished, the samples were sent to the Illumina Nova 6000 platform for high-throughput sequencing. Finally, the Cell

Ranger software (Version 4.0; 10x Genomics, USA) was employed to conduct alignments with the human reference genome (GRCh38).

Spatial transcriptome sequencing of osteosarcoma tissues

In this study, the 10x Visium CytAssist platform was employed for the spatial transcriptome sequencing of fresh - frozen (FF) tissue samples. After the tissue was excised, the residual blood and free tissue were thoroughly washed away with PBS solution, and the tissue was then placed into a 15 ml centrifuge tube containing RNA tissue preservation solution. All procedures were performed under strict aseptic conditions and completed within 30 minutes to minimize RNA degradation. Tissue samples were sectioned at 10 μm thickness using a cryostat microtome.

The tissue sections were sequentially fixed with methanol to preserve morphological integrity, followed by standard hematoxylin-eosin (HE) staining. High-resolution microscopy was employed to scan the stained sections for subsequent region of interest (ROI) selection. Following HE staining, tissue sections underwent decolorization treatment to ensure efficient penetration of hybridization probes into target tissue regions.

With the assistance of pathological experts, the areas on the HE - stained sections with a high density of tumor cells and rich infiltration of immune and stromal cells were selected as the ROI to explore the interactions among the components of the tumor microenvironment in the subsequent study.

The tissue sections were hybridized with the probes from the 10x Visium CytAssist Spatial Gene Expression for FFP kit. These probes contain specific sequences that can hybridize with the mRNA within the tissue and are marked with spatial barcodes for regional labeling, ensuring that the subsequent sequencing data can be traced back to their spatial locations.

Tissue sections were permeabilized using the permeabilization enzyme from the Visium Reagent Kit for a predetermined duration (12 minutes in this study), enabling the release of intracellular poly(A)+ mRNA. The released mRNA then bound to pre-immobilized oligonucleotide probes within designated capture areas on the slide, completing mRNA capture.

The captured mRNA was reverse - transcribed on the glass slide to synthesize cDNA, and spatial barcodes and UMIs were added to mark the spatial location information and the number of molecular copies. After the cDNA was amplified, the 10x Genomics Library Construction Kit (PN - 1000190) was used for library construction, including fragmentation, adapter addition, and PCR amplification.

The finally constructed spatial transcriptome library was subjected to paired - end sequencing (PE150 mode) on the Illumina NovaSeq 6000 platform to obtain high - coverage transcriptome data. The data were then spatially analyzed in combination with the spatial barcode information, ultimately generating a high - resolution gene expression map.
